# SuperNoder: a tool to discover over-represented modular structures in networks

**DOI:** 10.1186/s12859-018-2350-8

**Published:** 2018-09-10

**Authors:** Danilo Dessì, Jacopo Cirrone, Diego Reforgiato Recupero, Dennis Shasha

**Affiliations:** 1 Department of Mathematics and Computer Science, University of Cagliari, Cagliari, 09124 Italy; 2 Department of Computer Science, Courant Institute of Mathematical Sciences, New York University, New York City, 10012 USA

**Keywords:** Motifs discovery, PPI interaction network, Food-web network, Computational complexity, Network compression

## Abstract

**Background:**

Networks whose nodes have labels can seem complex. Fortunately, many have substructures that occur often (“motifs”). A societal example of a motif might be a household. Replacing such motifs by named supernodes reduces the complexity of the network and can bring out insightful features. Doing so repeatedly may give hints about higher level structures of the network. We call this recursive process *Recursive Supernode Extraction*.

**Results:**

This paper describes algorithms and a tool to discover disjoint (i.e. non-overlapping) motifs in a network, replacing those motifs by new nodes, and then recursing. We show applications in food-web and protein-protein interaction (PPI) networks where our methods reduce the complexity of the network and yield insights.

**Conclusions:**

SuperNoder is a web-based and standalone tool which enables the simplification of big graphs based on the reduction of high frequency motifs. It applies various strategies for identifying disjoint motifs with the goal of enhancing the understandability of networks.

## Background

Imagine describing a road map with words alone. The task would be difficult and unclear to most people. Networks provide a far better representation of any data representing interrelationships. However, because the size of modern networks (for example, in social science) can extend to thousands, millions, or even billions of nodes, networks themselves need to be abstracted for the sake of intelligibility and insight.

As in other disciplines, a way to reduce the size of the problem is to discover similar components and give them a common name. Linguists do this when they categorize parts of speech (noun, verb, adverb etc). Biologists do this when they group animals into species and families. In networks, we will do this by finding connected labeled sub-components that are isomorphic in label and topology. Formally, this entails finding common subgraphs or motifs that occur with a certain frequency.

Much research has proposed algorithms that aim at finding frequent motifs [1–5]. The motivation is usually to gain insights about metabolic and protein-protein interactions, ecological food-webs, social networks, collaboration networks, information networks of interlinked documents and products [6–14].

Most of this work does not distinguish between motifs that overlap and motifs that do not. However, this distinction can be critical for understandability. For example, households are a convenient abstraction in social graphs because they are disjoint whereas friendship motifs do not tend to be. For networks whose motifs are not naturally disjoint, identifying disjoint motifs may help to understand network structure (e.g. cliques in friendship networks). One work that has done this is [15] which showed algorithms to find edge-disjoint motifs in unlabeled networks. Our work focuses on node-disjoint motifs (which share neither nodes nor edges) in labeled networks. The usefulness of labels is intuitive as we will see in our examples and node-disjoint motifs are readily decomposable. We also present promising algorithms to make this process reasonably fast even for sizeable networks.

**Fig. 1:**
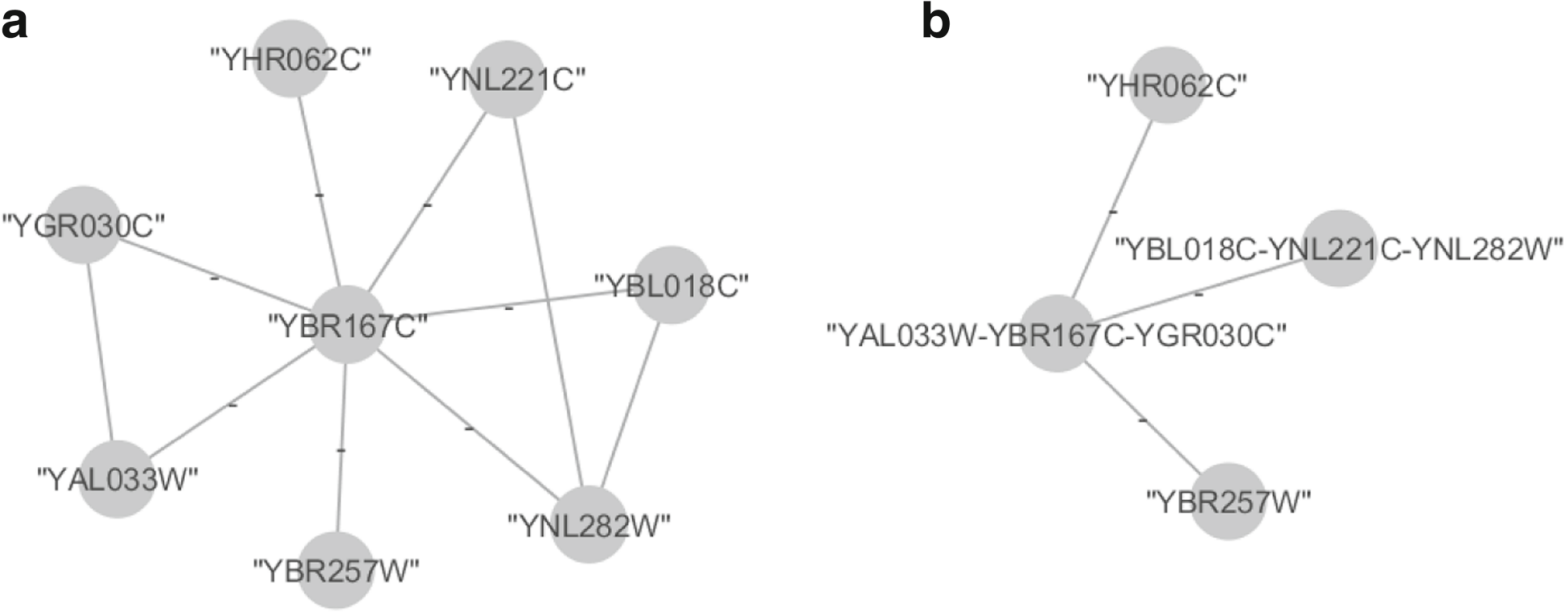
Example of motifs collapsed into supernodes in a Protein-Protein Interaction network. **a** The original nodes of the network. **b** The new nodes of the network after two motifs of size three have been collapsed

Once disjoint motifs of a certain size *k* have been identified, each such motif can be collapsed into a *supernode*, which is a single node that inherits all the connections and properties of the motifs. This procedure can be performed recursively in order to find motifs on graphs consisting of a combination of nodes and super-nodes. Figure 1 shows an example where motifs have been collapsed into supernodes.

Thus, our tool SuperNoder finds disjoint motifs on a base graph G1, reducing G1 to a new graph G2, and then recursively repeats the procedure to find G3, G4, and so on. SuperNoder attempts to find the most possible disjoint frequent motifs of a given size in a target network in each stage of the process. We present several techniques to achieve this goal.

Orthogonally, the SuperNoder tool can take input nodes at different layers in a label hierarchy. For example in phylogeny, there is a hierarchy of species, genus, family, kingdom. Relationships that may be obscure at a low level may be clearer at a high level (e.g. felines eat rodents).

This paper makes three contributions:

- Efficient algorithms to find disjoint supernodes in labeled networks, including networks already containing supernodes, yielding a recursive algorithm.
- A tool incorporating these algorithms that is free to the community.
- Example applications to show the usefulness of the approach.

Frequent (based on the possibly overlapping F1 measure) motifs have been shown to give insights in regulatory [16], food-web [17–19], and social science [20, 21] networks. Reduction methods aim at minimizing the loss of information while maximizing the understandability, often establishing which components are less interesting for the behavior of networks. Recent studies have focused on finding high-order clusterings [22, 23]. However, most of this research has focused on modeling graphs without considering node labels, despite the fact that many networks have them. Moreover, they usually consider overlapping motifs, therefore, a single node can belong to several patterns, making further analysis (and understandability) difficult.

An early compression graph method was proposed by [24] where the authors show how finding substructures and merging them in vertexes for compressing data. Our approach builds on theirs, but their approach does not find all substructures that occur nor does it attempt to find the most highly repetitive subgraphs which are the best candidates for capturing subgraph regularities.

Our work also draws inspiration from [15] where the authors propose two methods to find disjoint motifs under the F2 frequency measure (where two graphs are disjoint if they do not share a common edge). First, they propose a method to find motifs based on a small set of patterns, and then give methods to find non-overlapping motifs solving the Maximum Independent Set (MIS) problem. They invented their own method for finding frequent motifs and did not choose to compare their method with state-of-the-art motif-finding techniques [25–30]. By contrast, we have chosen to base our approach on the motif-finding algorithm of [25] because of its simple implementation and promising results [31]. As in [15], the second phase of our algorithm uses an *overlap graph*, and we have explored some heuristics to deal with larger *overlap graphs* beyond what they used.

While we do contribute algorithms for finding *disjoint* motifs given a collection of already found motifs, we do not advance the state of the art in finding the motifs themselves. Instead, our work builds on top of an existing overlapping motif finding algorithm which has been compared and studied many times in literature [31].

The remainder of this paper is organized as follows. “Implementation” section describes the proposed approach. “Results” section describes the biological datasets we have used, shows an example application of SuperNoder to the yeast network, and analyzes both the performance and quality of SuperNoder on real networks. “Conclusions” section gives perspectives on the problem and future directions. “Availability and requirements” section reports where the tool can be found with its essential requirements. Finally, “Abbreviations” section lists abbreviations we use in the paper.

## Implementation

Labeled networks or graphs are formally characterized by a triple *G*=(*N*,*E*,*L*) where *N* denotes a set of nodes, *E* denotes a set of edges (pairs) *e*=(*n*_*i*_,*n*_*j*_)∈*N*, and *L* is a mapping from *N* to some set of labels. Edges represent an application-dependent relationship. For instance, an edge may connect two nodes representing people if the people are friends.

We say that a graph is *undirected* if every edge from *n* to *n*^′^ implies the existence of an edge from *n*^′^ to *n*. Otherwise the graph is said to be *directed*. A *subgraph* is a *connected* component *G*_*S*_=(*N*_*S*_,*E*_*S*_) such that *N*_*S*_⊆*N* and *E*_*S*_⊆*E* if there exists a path from each *n*_*i*_∈*N*_*S*_ to each *n*_*j*_∈*N*_*S*_. A *k*−*subgraph* is a subgraph with *k* nodes.

Two subgraphs *S*_1_,*S*_2_ are *isomorphic* if (i) there exists a bijective function *f*:*N*_*S*1_→*N*_*S*2_ such that for each pair (*n*_*i*_,*n*_*j*_)∈*E*_*S*1_⇔(*f*(*n*_*i*_),*f*(*n*_*j*_))∈*E*_*S*2_ and (ii) for all *k*, the label of *n*_*k*_. *L*(*n*_*k*_) is the same as *L*(*f*(*n*_*k*_)). To count the number of occurrences of a given subgraph, three different measures can be used [32]. The first measure, named F1, is the count of each subgraph regardless of whether it overlaps with others. The second one, named F2, avoids overlaps of subgraphs if they share at least an edge (or equivalently a connected pair of nodes). The last one, named F3, requires that two subgraphs share no nodes. F3 is therefore, the most strict criterion of disjointness (and is the one used in this paper). We define the *frequency* of a subgraph *S*_1_ in *G* as the number of occurrences of *S*_1_ in *G*. We call subgraphs *k*−*motifs* if they have *k* nodes and occur over a threshold *t* using the F1 measure.

The SuperNoder pipeline consists of the following steps:

1. Solicit a size *s* from the user corresponding to the number of nodes each motif should have.
2. Solicit a threshold *t* from the user corresponding to the number of times that a motif should be present to be considered. (In the future, we may add specific shapes of motifs or specific motifs labels, as further filters in addition to threshold.)
3. Search for all possible motifs in the input network meeting threshold *t*, using the F1 measure (i.e. allowing overlaps). Call that set *M*.
4. Search for the maximum number of non-overlapping motifs from *M*.
5. Collapse non-overlapping motifs into supernodes.
6. Repeat steps 2 through 5 until satisfied.

In this section we provide details of our tool for accomplishing these tasks.

### Input network and motifs finding

SuperNoder requires two series of data as an input:

- A list of node rows, where each row represents a node by means of a unique *ID* and a *label* separated by a blank space.
- A list of edge rows, where each row consists of two node *IDs* separated by a blank space.

SuperNoder uses the *Randomized Enumeration* algorithm [25] for the purpose of motif finding. The result of the algorithm is a set of all possible undirected motifs in the network, allowing overlaps.

### Motif count and thresholding

To count motifs, we implemented a function to compute isomorphisms between subgraphs similar to the one of Cordella and colleagues [33]. First, the algorithm takes the labels of subgraph nodes and counts how many nodes have the same label. Second, for each label it computes the sum of in-degrees and the sum of out-degrees (i.e. for each node label, it computes *l*_*n*,*i*,*o*_, where *n* is the number of nodes with label *l*, *i* is the sum of in-degree of nodes with label *l*, and *o* is the sum of out-degree of nodes with label *l*). Finally, it sorts these labels using the lexicographic order and computes their hash. If the number of subgraphs having hash value *h* is greater than the user-given threshold *t*, then all such subgraphs are checked to see how many are in fact isomorphic. If, after the check, the number is greater than *t*, then those subgraphs pass the initial filter to be a motif and thus belong to the “frequent motif set”. Thus the frequent motif set may contain different topologies, e.g. at least *t* stars of size *s*, at least *t* paths of length *s*, and so on.

### Finding disjoint motifs

Our methods to find disjoint motifs, given the potentially overlapping frequent motif set, uses the concept of an *overlap graph*. An *overlap graph* is a pair (*M*,*E*) where *M* is the set of motifs and there is an edge between motif *m*1 and motif *m*2 if they share at least one node in the original graph. (In the case of recursive reduction, the original graph at reduction i is the one produced from the graph at reduction i-1, containing both normal nodes and supernodes.)

We briefly present an overview of our heuristics for finding disjoint motifs here (Table 1), but the full pseudo-code is available in the github site containing the SuperNoder source code as well.

**Table 1:** Summary of the characteristics of the heuristics

Heuristic	Overlap		Order	Random	Sampling
ID	graph	Ramsey	by degree	approach	approach
H1	-	-	-	V	-
H2	V	V	-	-	V
H3	-	-	V	V	-
H4	-	-	V	V	-
H5	V	-	V	V	V

The symbol V indicates that the heuristic exploits that characteristic, - if not. H1 = Greedy Elimination. H2 = Ramsey. H3 = Ranked Elimination. H4 = Ranked Replacement. H5 = Sampled Ranked Elimination

**H1 (Greedy Elimination)**. This simple but effective heuristic finds disjoint motifs by using a Maximal Independent Set technique. Given the frequent motif set *M* and a user-given parameter *n*, randomly shuffle the potentially overlapping motif instances from the frequent motif set *M*. For each motif instance *m*, if the motif instance overlaps no other motif instances of *M*, then output it. Otherwise remove it and all its edges from the overlap graph. Because this approach is naively greedy, SuperNoder tries *n* (parameter given by the user) different random shufflings to try to obtain the greatest number of disjoint motifs.

**H2 (Ramsey)** Heuristic-2 exploits both sampling and the Ramsey method whose functions can be seen in [34]. Given the list of motif instances *M* and a number *k*, the heuristic (i) takes disjoint subsets of size *k* from *M* and constructs the induced subgraph of the overlap network from each subset. (ii) On each subgraph, it performs the Ramsey algorithm obtaining a *MIS*_*subgraph*_. (iii) Then, it merges all *MIS*_*subgraph*_*s* into a reduced list of motif instances which takes the role of *M*. The algorithm repeats steps (i) through (iii) until there are no more overlaps and outputs the resulting set of motifs.

**H3 (Ranked Elimination)**. Heuristic-3 assigns to each (possibly overlapping) motif instance *m* a degree equal to the sum of degrees of the nodes in *m* ignoring the edges between nodes in *m* (i.e. the sum of the degrees of the nodes in *m* pertaining to edges that connect to nodes outside *m*). The algorithm then orders the motif instances in ascending order of degree so calculated, forming a list called *MotifDegree*. For each node *n* in the original graph, find the first motif instance in *MotifDegree* and discard all other motifs in *MotifDegree* containing *n*. This process yields a new list called *PotentialSuperNodes*. Then traverse this *PotentialSuperNodes* list, preserving motif instances having no overlaps and deleting motif instances that have higher degrees when there are overlaps.

**H4 (Repeated Ranked Elimination)**. This approach is an improvement over H3, because H3 misses some motif instances when one or more overlapping motif instances are removed and the nodes of the removed motif instances then have no chance to be included in any other motif instances. Given as input the list of motif instances *M* found using the *Randomized Enumeration* method seen above, build the *MotifDegree* list as in Heuristic-3. For each node *n*, the motif instance *m*∈*MotifDegree* with the lowest degree that contains *n* is copied to a list of potential supernodes, called *PotentialSuperNodes*. All the motif instances in *PotentialSuperNodes* with no overlaps are considered valid. Then, for each pair {*m*^′^,*m*^′′^} of overlapping motif instances in *PotentialSupernodes*, discard the motif instance with the higher degree. Continue until there are no more motif instances. Now consider all the nodes *N*_*orphan*_ that are not in any disjoint motif instance found so far and consider motif instances based on the F1 measure that apply to nodes of *N*_*orphan*_. Repeat the above procedure to generate more disjoint motif instances. Repeat until there are no more nodes in *N*_*orphan*_.

**H5 (Sampled Ranked Elimination)**. This heuristic unifies sampling with the overlap graph approach. After the sampling is done as for the Ramsey algorithm, the heuristic constructs an *overlap graph* on the surviving motif instances. The heuristic considers the motif instances in ascending order by degree in the *overlap graph*. If a motif instance has no edges, then put it in the result. If a motif instance *m*1 has an edge with another motif instance *m*2, then remove the motif instance with the largest degree.

### Network reduction

After the non-overlapping motif instances have been found, each one is collapsed into a supernode, preserving the external connections of the original nodes of motifs. The label of each supernode is the concatenation of labels of its member nodes in alphabetical order. The new network can be saved as an output using the same format as the input network and the whole pipeline can be iterated on it.

## Results

### The test networks

We demonstrate SuperNoder on three different labeled biological networks:

- A food-web subnetwork of Florida bay network^1^ [35] with 93 nodes and 960 edges.
- A Protein-Protein Interaction (PPI) network of yeast^2^ [36] with 2361 nodes and 7182 edges.
- A PPI network of Arabidopsis^3^ [37] with 18167 nodes and 10928 edges.

**Food-web network**. The original nodes have labels that represent animals or plants (e.g. *predatory chanodichthys*, *dinoflagellates*, *coral bryaninops*, etc.). We have mapped the network using a taxonomy^4^, retrieving for each node *genus*, *family*, *order*, *class*, *phylum*, and *kingdom*. From the original network we have removed species that did not have higher phylogenetic categories.

**Protein-Protein Interaction networks**. In a Protein-Protein Interaction (PPI) network, each node represents a different protein. For the higher-level categorization of PPI networks, we have employed the ontology annotations available at this link^5^. First, we have retrieved the Gene Ontology (GO) term that belongs to Biological Processes (BPs) and that has the lowest (i.e. most empirically based) evidence code for each protein. Second, we have traversed the ontology *go-basic*^6^ starting from each GO term in our network to the GO term which represents all Biological Processes. Since each GO term can have more than one parent, we have chosen the GO term with the lowest (i.e., most conclusive) evidence code going up in the hierarchy. More precisely, given a label of a node *l*, we retrieve a GO term *g* with the lowest evidence code. Let {*g*_1_,*g*_2_,...,*g*_*n*_} be the parents of *g*, then we choose the *g*_*i*_ with 1≤*i*≤*n* with the lowest evidence code, building a hierarchy *l*,*g*,*g*_*i*_. Then, we repeat the same operation as long as the GO term which represents all Biological Processes (BPs) has not been yet reached. In doing so, we have built a taxonomy that can enable the analysis of protein functions.

### Use case

In the analysis of biological networks, interactions often occur between proteins of the same class [38]. SuperNoder can find these relations when high level functional classes are considered, highlighting frequent related processes and simplifying their identification.

To show how SuperNoder may help to simplify networks, we focus on the yeast network, and explain how higher levels of the Gene Ontology (GO) terms enable the abstraction of protein functions allowing SuperNoder to reduce the network complexity. The motivation is simple: at a lower level in the hierarchy of GO terms there may be no motifs that occur more than *t* times for a moderately large *t*. At higher levels, there might be. In the example, the yeast network has been mapped onto five levels of the GO terms hierarchy. To be considered a motif, a subgraph has to occur at least 50 times, i.e. with threshold *t*=50.

**Fig. 2:**
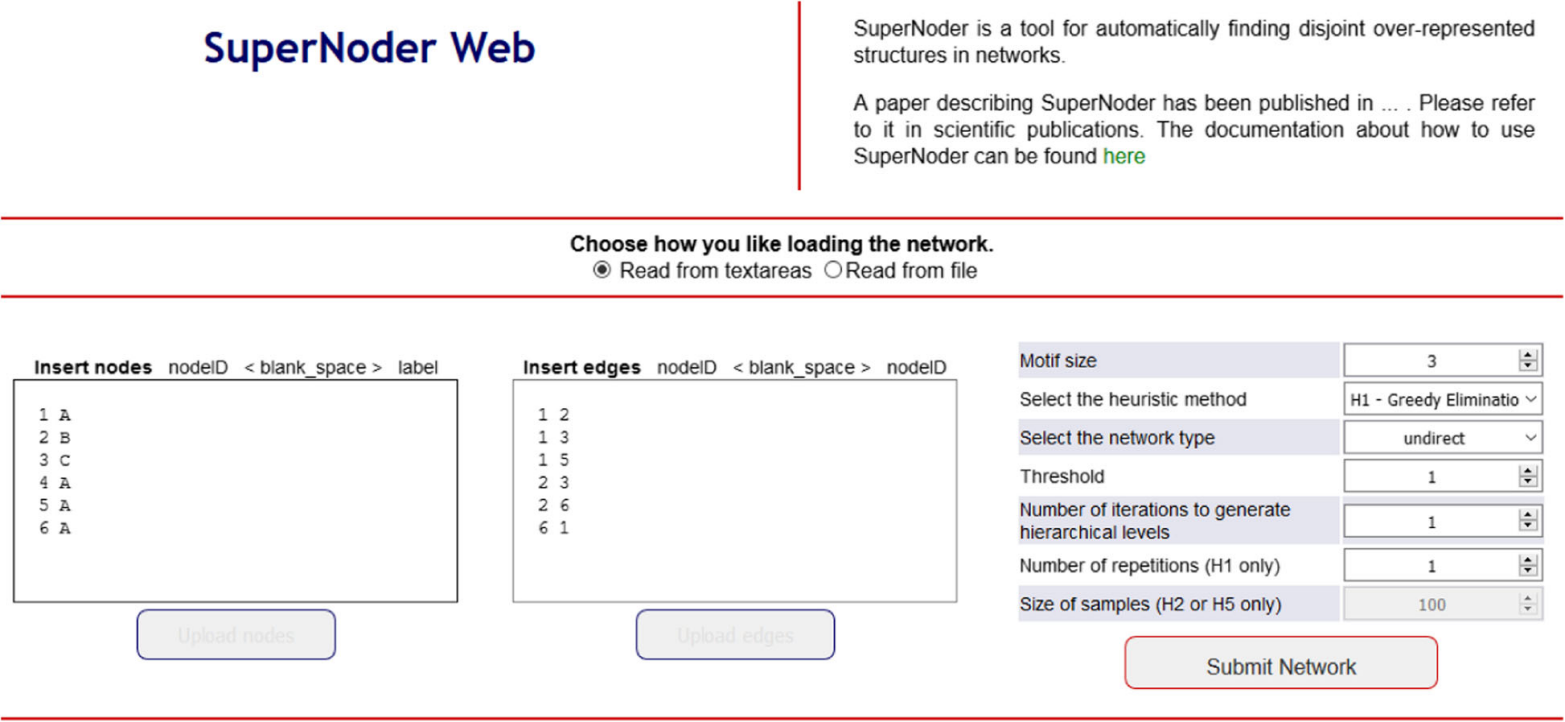
An example of four supernodes built using SuperNoder with motifs of size three on the yeast network. From left to right, labels of original nodes, labels of the fifth level hierarchy, labels of the third level hierarchy. On the third level, many proteins share the same pattern and these patterns are often disjoint

Figure 2 shows a motif of size three in each row that are mapped on the base level (gene labels), the fifth-level (L5) and the third-level (L3) hierarchy labels (i.e. in ascending order of abstraction). More motifs appear at higher levels in the hierarchy (i.e. first on L5 and then on L3 levels). In fact, with L5 labels the triples in row 2 and row 3 are isomorphic. When L3 labels are used, all triples are isomorphic, thus becoming relevant motifs. Those triples are collapsed into supernodes thus forming a new simplified network. Supernodes indicate proteins that belong to the same class helping biologists with the analysis of basic interactions.

As a specific case study, focus on motifs composed of proteins (*YNL306W, YDR175C, YBR251W*) and (*YGR156W, YKR002W, YLR115W*). Analyzing the network on the base labels, there are not supernodes, since they do not show common features in the labeled graph. Already at lower hierarchical levels (i.e. L5), the motifs GO terms are abstracted into functions, viz, *macromolecule biosynthetic process* and *cellular macromolecule metabolic process* respectively. At hierarchical level L3, the proteins in this example have the label GO:0071704 which indicates that their proteins are related to *organic substance metabolic process*. At that level, we find out that *organic substance metabolic process* (GO:0071704) covers an important role into the yeast network, and that is mainly composed of *macromolecule biosynthetic process* (GO:0009059), *cellular macromolecule metabolic process* (GO:0044260) and *protein metabolic process* (GO:0019538). This shows an example of how our tool can help biologists understand the behavior of proteins (with frequent motifs) belonging to the same class.

**Fig. 3:**
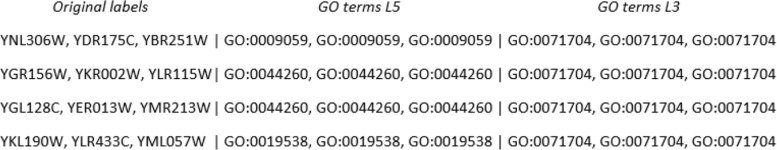
Figures show samples of the yeast network with 25 nodes mapped with original and GO terms labels of our yeast GO hierarchy, and where supernodes have been found by means of SuperNoder. **a** Original network with 25 nodes and 83 edges. **b** Network reduced on low level GO terms hierarchy (19 nodes and 67 edges). **c** Network reduced on high level GO terms hierarchy (11 nodes and 43 edges)

The higher the hierarchy levels, the larger the number of relevant motifs that can be used to further reduce the current network (an example of this behavior can be observed in Table 2). In addition, higher level labels enable higher thresholds, sometimes leading to the discovery of very frequent motifs. For example, connections of proteins in Fig. 3a do not show functionalities but those become evident at higher hierarchical levels 3b and 3c. For example, the frequent relation between proteins which have *GO:0044237, GO:0044237, GO:0044237* as GO terms that are showed in Fig. 3c are only detectable at that level of the hierarchy. Finally, images 3b and 3c show that the reduction at a high level of abstraction enables a better understandability of the network.

**Table 2:** An example of a hierarchical exploration of the yeast network

	th	Original	L5	L4	L3	L2	L1
Motifs	25	0	290	292	319	377	389
Nodes	25	2361	1781	1776	1607	1583	1333
Edges	25	7182	5234	5305	5018	5020	5322
Motifs	50	0	240	236	304	388	390
Nodes	50	2361	1841	1889	1585	1361	1581
Edges	50	7182	5339	5429	5029	5347	4990

The table reports the number of found motifs, the number of nodes and edges, when the network is mapped to different levels of the GO terms hierarchy and then reduced. At higher levels (L1 is higher level than L2 etc) more motifs pass the threshold

### Performance

In this section, we report the time performance, the number of disjoint motifs and the reduction ability of our heuristic algorithms. The time performance is based on the wall clock time required for the execution of the heuristics on all relevant motifs. The number of disjoint motifs is the number of motifs found by each algorithm. The reduction ability is the extent of reduction of networks. All experiments have been performed considering motifs with size = 3 and size = 5 (i.e. having three nodes in the original graph and three nodes or supernodes after each step of the recursion). H1 has been performed with five shufflings. H2 and H5 adopted subsets of the overlap graphs consisting of 1000 motif nodes. In our simulations, we chose different thresholds in different networks, as shown in Tables 3 and 4. The reason is that certain thresholds make no sense for certain networks. For example, a threshold of 100 for our food-web network is meaningless because no motifs occur that frequently.

**Table 3:** Rows list the number of all motifs, the threshold applied in our experiments and the number of motifs that meet that threshold when L3 labels are considered and motifs have size 3

Network	N motifs	Threshold	N repetitive motifs
Food-Web	20283	5	5085
Yeast	96444	50	49294
Arabidopsis	268437	100	155185

**Table 4:** Rows list the number of all motifs, the threshold applied in our experiments and the number of motifs that meet that threshold when L3 labels are considered and motifs have size 5

Network	N motifs	Threshold	N repetitive motifs
Food-Web	26841	5	407
Yeast	188733	50	11550
Arabidopsis	425895	100	14474

#### Food-web network

**Fig. 4:**
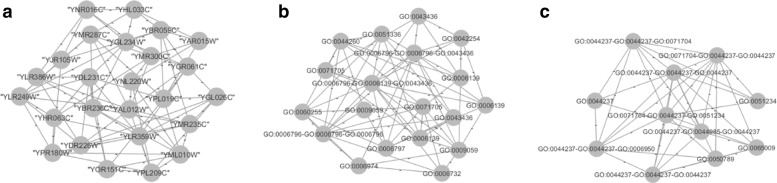
SuperNoder heuristics performance on the food-web network considering motifs of size 3 and 5 in terms of (**a**) the number of unique motifs found (**b**) the running time

Figure 4 reports the performance of the heuristics applied on the food-web network. In this case, heuristics H1, H2 and H5 which exploit repetitive random approaches (H1), sampled *overlap graph* (H2 and H5), and H4 show better performance than others in finding disjoint motifs. Heuristics H3 shows a poor reduction factor on this network. The reason is that there are many motifs with the same sums of degrees, so degree-based heuristics do not work well. Heuristic H1 is the fastest. This holds regardless of motif size. In fact, overall, heuristic H1 is both fast and has a good reduction factor.

#### Yeast network

**Fig. 5:**
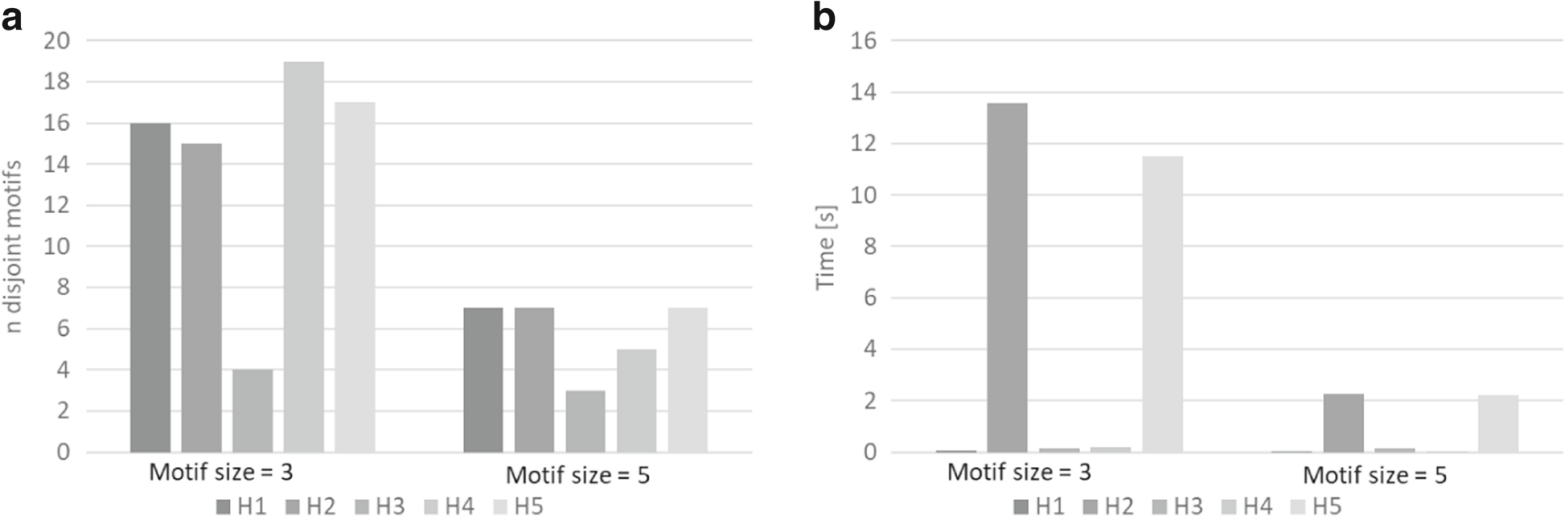
SuperNoder heuristics performance on the yeast network considering motifs of size 3 and 5 in terms of (**a**) the number of unique motifs found (**b**) the running time

Figure 5 shows the performance on the yeast network. In contrast to the food-web network, heuristics H2 and H5 based on the sampled *overlap graph* do not obtain the best reduction factor. In this case, heuristic H4 enjoys a greater reduction factor. Although heuristics H2 and H5 can find a large number of disjoint motifs, they require excessive time to find a solution, hence, their use on a network of this size might be avoided. The heuristics H1 and H3 are still the fastest.

#### Arabidopsis network

**Fig. 6:**
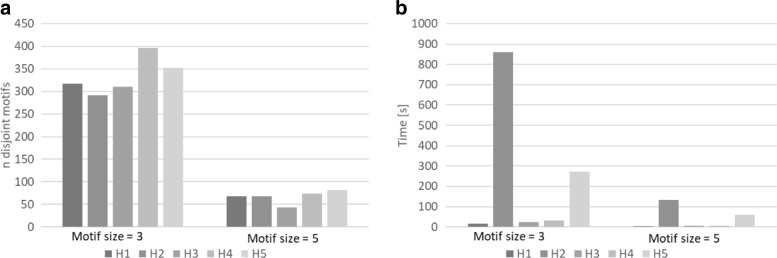
SuperNoder heuristics performance on the Arabidopsis network considering motifs of size 3 and 5 in terms of (**a**) the number of unique motifs found (**b**) the running time

Experimental results on arabidopsis networks (see Fig. 6) are similar to those on the yeast network and the same considerations hold. Note that the arabidopsis network is a Protein-Protein Interaction network like the yeast network but is very different in term of size.

#### Observations from the Experiments

Heuristic H1 achieves the best time performance and finds a large number of disjoint motifs though not always the maximum number. Heuristic H4 which is slower can sometimes find more disjoint motifs so should be considered if time is available. The size of motifs and the threshold also matter. Larger motifs entail the processing of more data, but there are fewer repetitive motifs (i.e. motifs that exceed the threshold) so the overall time is sometimes less.

In summary, heuristic H1 shows good performance on all types of network since its greedy approach is fast. The resulting reduction may not however be best. Heuristics H2 and H5 which employ sampling are useful for those networks whose overlap graphs are very large. The size of samples can be chosen according to the available computational resources to balance the execution time and memory use. Heuristic H2 should show better reduction performance than H5 when there are few distinct motifs degree values. By contrast, H3 and H4 should be useful for all those networks that have many distinct motifs degree values, because motifs having less probability to overlap are detected faster.

#### Reduction

**Fig. 7:**
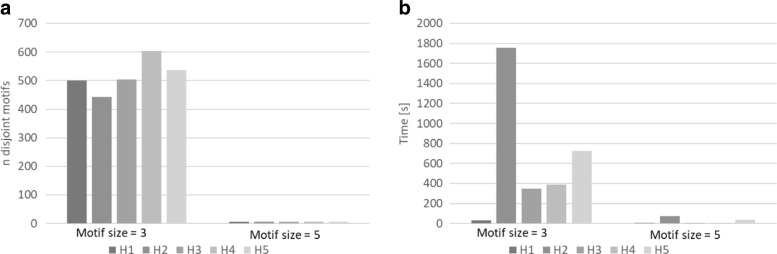
Reduction performance on five iterations on the food-web network (**a**) motifs of size 3 without threshold (**b**) motifs of size 3 with threshold (**c**) motifs of size 5 without threshold (**d**) motifs of size 5 with threshold

**Fig. 8:**
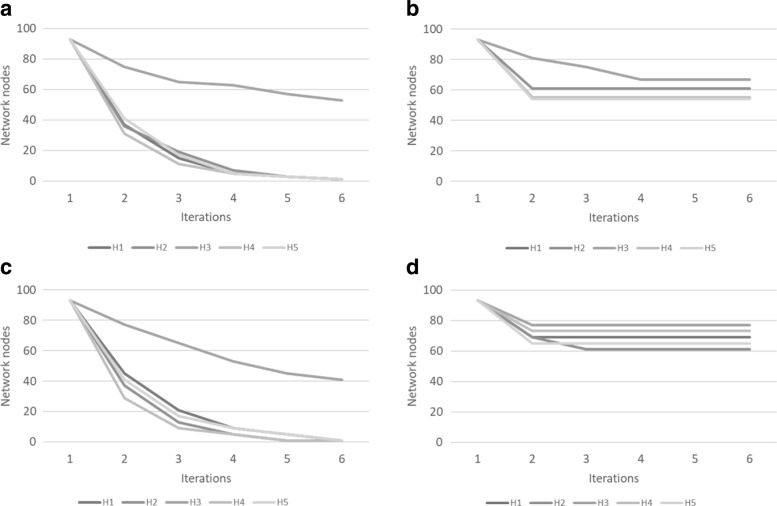
Reduction performance on five iterations on the yeast network (**a**) motifs of size 3 without threshold (**b**) motifs of size 3 with threshold (**c**) motifs of size 5 without threshold (**d**) motifs of size 5 with threshold

**Fig. 9:**
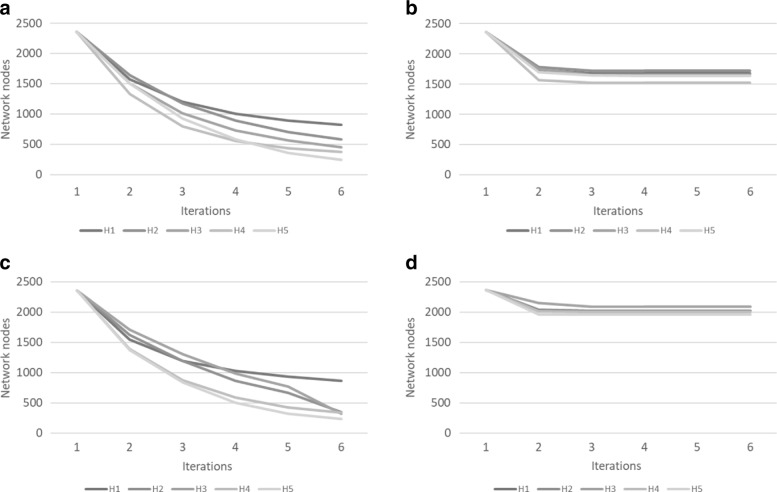
Reduction in size of the food web network mapped on the species order (e.g. *kingfisher* mapped on *coraciiformes*). **a** The original network. **b** The network reduced after 1 iteration. **c** The network reduced after 2 iterations

Figures 7 and 8 show the extent of graph reduction on the food-web and yeast networks respectively. Unsurprisingly, lowering the threshold generates more F1 motifs, increasing the number of F3 motifs and reducing the network size. In our example networks, after a few iterations, the networks are no longer reduced. When this plateau-ing happens depends entirely on the data. In addition, the threshold and the motif size both affect the reduction factor, because a small motif has a higher probability of occurring more often (see Tables 3 and 4). This is well illustrated by our tests where motifs of size 3 show a greater reduction than motifs of size 5. For an illustration of the extent of reduction, consider Fig. 9 where (a) shows the original food web network, (b) after one iteration and (c) after two iterations.

### Tool description

**Fig. 10:**
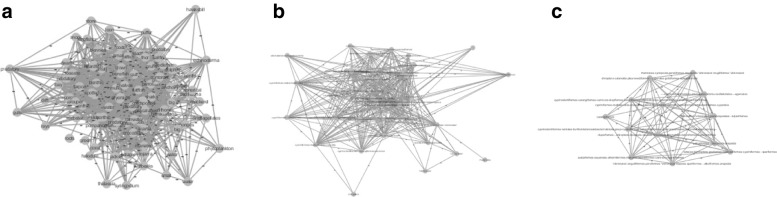
SuperNoder web application

Figure 10 shows the graphical interface of SuperNoder that users without programming skills can adopt to analyze networks. On the left, users can use a panel to create nodes, in the center there is one panel to create edges, and, on the right, a list of parameters the user can set. With the first option users can choose the size of motifs they are interested in. The minimum value is 3. The next option is related to the heuristic that should be employed to find disjoint motifs. The user can also choose the type of network: *direct* or *undirect*. The fourth parameter is the threshold which represents the minimum value each motif should meet to be considered over-represented (it corresponds to the threshold *t* of the SuperNoder pipeline algorithm). The last required parameter is the number of iterations. In addition, if the user selects the H1 heuristic, he/she can set the number of repetitions to be executed, specific for H1. If the user selects either the H2 or H5 heuristic, he/she can also choose the size of samples. When the *Submit network* button is clicked, the SuperNoder pipeline will be run and results will be printed and shown online (but not saved anywhere).

The output consists of two sections (nodes and edges) for each chosen iteration using the same input format. Supernodes are indicated by the tag *#supernode*.

The code has been developed in Python 3.6 using NetworkX^7^ library. SuperNoder functionalities operate on graphs using the standard NetworkX format. The web interface is provided by a python server which runs on a Docker^8^ container. Last but not least, SuperNoder is hosted on a GitHub^9^ page and distributed as a Docker file with the source code freely available under GPLv3 License.

## Conclusions

SuperNoder enables the simplification and compression of graphs based on high frequency motifs. By identifying disjoint motifs, SuperNoder enhances understandability as the network is reduced. This paper describes and compares various algorithms on real networks, both to show the benefits of the approach and to find high-performing algorithms. SuperNoder has been developed in Python, it can either be installed on local machines or used through its online web interface. Future work includes enhancing performance yet further by using Graphical Processing Units.

## Availability and requirements

**Project name**: SuperNoder

**Project homepage**: http://glab.sc.unica.it/supernoder/

**Github link**: https://github.com/danilo-dessi/SuperNoder-v1.0https://github.com/danilo-dessi/SuperNoder-v1.0

**Operating system(s)**: Platform independent

**Programming language**: Python

**Other requirements**: Docker

**License**: GPLv3.

**Any restrictions to use by non-academics**: nothing.

## Abbreviations

MIS: Maximum independent set
PPI: Protein-protein interaction

## Footnotes

1
https://snap.stanford.edu/data/Florida-bay.html

2
http://vlado.fmf.uni-lj.si/pub/networks/data/bio/yeast/yeast.htm

3
http://interactome.dfci.harvard.edu/A_thaliana/index.php?page=download

4
https://ftp.ncbi.nlm.nih.gov/pub/taxonomy/

5
http://www.geneontology.org/page/download-annotations

6
http://www.geneontology.org/page/download-ontology

7
https://networkx.github.io/

8
https://www.docker.com/

9
https://github.com/danilo-dessi/SuperNoder-v1.0
